# Sensory perception and consumer acceptance of commercial and salt-reduced potato crisps formulated using salt reduction design rules

**DOI:** 10.1016/j.foodres.2022.111022

**Published:** 2022-05

**Authors:** Katherine E. Hurst, Louise Hewson, Ian D. Fisk

**Affiliations:** a Division of Food, Nutrition and Dietetics, University of Nottingham, Sutton Bonington Campus, Loughborough LE12 5RD, United Kingdom; b The University of Adelaide, North Terrace, Adelaide, South Australia, Australia

**Keywords:** Salt reduction, Saltiness perception, Potato crisps, Mouth behaviour, Texture

## Abstract

Successful salt (NaCl) reduction strategies are required to reduce the salt content of snacks while maintaining saltiness perception and consumer acceptance. Previous research suggests that particle physicochemical design rules (small particle size, low density, low hydrophobicity, optimised particle shape) can be leveraged to produce salt particles that enhance saltiness perception. This study aimed to validate these design rules by applying optimised model salts to unsalted potato crisps at a 30% reduced salt content to produce prototype products. A selection of commercial products were also chosen to represent the salt content and crisp style of the broader market, with the aim to investigate the potential of other salt reduction strategies including; direct salt removal without compensation for loss of salt content and increasing time in mouth, while exploring the impact of consumer mouth behaviour type on consumer product preference. Nine products varying in salt content (6 standard, 1 crinkle-cut, 1 thick-cut batch-fried, 1 baked reconstituted potato) were subject to descriptive sensory analysis with a trained panel (n = 11). A subset (seven products) were assessed for consumer acceptance (n = 93). A salt reduction of 30% was achieved while maintaining saltiness perception and consumer acceptance using model salts, while direct removal of salt without perceptual impact was only achievable by 15%. To investigate key drivers of liking, consumers were segmented based on product liking and mouth behaviour. Results suggested that whilst salt content was the primary driver, specific texture profiles were polarising. However, mouth behaviour had minimal influence on preference. These results validate previously described physicochemical design rules for developing novel salt particles for salt reduction and inform ingredient design for the food and flavour industries.

## Highlights

• Optimised model salt particles were selected based on physicochemical design rules.
• Model salts applied to potato crisps were evaluated alongside commercial products.
• Model salts allowed a 30% salt reduction without impacting saltiness or acceptance.
• Only 15% salt could be directly removed without impact on perception and acceptance.
• Salt content drives liking, and liking of texture profiles were polarising.

## Graphical abstract

**Figure:**
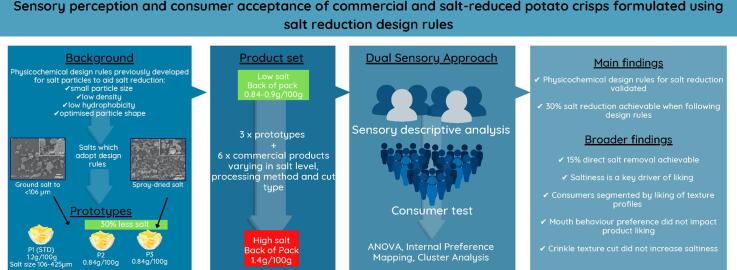

## 1 Introduction

Cardiovascular diseases (CVDs) are the leading cause of death worldwide, contributing to one-third of global deaths in 2019 (World Health Organisation, 2021). CVD is a group of disorders associated with the heart and blood vessels, of which a diet high in sodium chloride salt (NaCl) is one of the major risk factors (World Health Organisation, 2021). In addition to the many preventable deaths caused each year, CVDs also place a substantial financial burden on the global economy through increased healthcare costs (Public Health England, 2019). It is recommended that adults consume no>5 g NaCl/day (World Health Organisation, 2018); however, average salt consumption still exceeds these levels in most countries, e.g. 8 g/day in the United Kingdom, 9 g/day in Australia and USA, and 14 g/day in China (Thout et al., 2019, Zhou et al., 2019).

Since the highest contributor to salt in our diet is processed foods (Gibson et al., 2000), most developed countries have continuing voluntary or mandatory salt reduction targets (Webster et al., 2011) to encourage food manufacturers to reduce salt in their products. Removal of NaCl salt is difficult due to its many functionalities, for example: imparting flavour, controlling yeast growth and fermentation rate, improving product texture, reducing spoilage through control of water activity, contributing to food preservation, and increasing water-binding capacity (Man, 2007). For savoury snacks specifically, it provides a salty taste to an otherwise bland base, enhances flavour, provides a desirable product texture to expanded or reconstituted snacks, acts as a carrier for some seasonings, and may help to improve seasoning flowability (Man, 2007). Since salty taste is a key driver of consumer acceptance in many processed foods (Li et al., 2015), and direct removal is highly problematic due to its multifaceted role, significant research is required to enable food manufacturers to meet salt reduction targets without compromising consumer acceptance. Salt reduction strategies include: direct removal (partial removal of salt without compensatory strategy), salt substitution, leveraging cross-modal interactions for saltiness enhancement and modification of the product or salt particle to increase the availability of sodium and dissolution rate in saliva (Busch et al., 2013, Kilcast and Angus, 2007, Kuo and Lee, 2014). Salt reduction strategies should depend on the functional property of salt in that particular product. The use of direct removal without an attempt to mitigate the loss of salt content can be achieved up to 20% in certain applications, including bread and dairy products (Drake et al., 2011, Jaenke et al., 2017, La Croix et al., 2015, Levings et al., 2014, Mueller et al., 2016), with even up to 40% reduction being suggested as possible (Torrico et al., 2019). This is possible since partial reduction in salt concentration in a product can go unnoticed by the consumer. However, direct removal risks consumers rejecting the low salt product and ultimately choosing an alternative product that provides the desired saltiness. In addition, consumers may compensate for the loss of saltiness by adding their own salt (Zandstra et al., 2016). In order to combat this issue and reduce the risk of consumer rejection of any new salt-reduced formulations by direct removal, a common approach is to use reduction by stealth, where gradual reduction over time can result in considerable reductions over an extended time period (Kilcast & Angus, 2007). Previously, in cereals and bread, a 33% and 25% reduction in NaCl salt content over time was achieved, respectively (Kilcast & Angus, 2007). Although successful, the approach required 7 years to ensure that the reduction remained unnoticed by consumers and still requires a concerted agreement within the specific product industry; to avoid the risks associated with the direct salt removal method, as mentioned. Potassium chloride (KCl) has been used to reduce salt by replacing NaCl salt up to 30% in some products, including potato crisps (Kongstad and Giacalone, 2020, Mueller et al., 2016, Torrico et al., 2019). However, undesirable metallic and bitter flavour notes associated with increasing KCl content limit this approach (Sinopoli & Lawless, 2012). In potato crisps, it was also found that consumers perceive the use of KCl as a replacement of NaCl salt as less healthy and may therefore reject this change in reformulation (Kongstad & Giacalone, 2020). Another way to mitigate the loss of salt content during reduction is to increase sodium availability and dissolution either by altering product matrix composition (Kuo and Lee, 2014, Lawrence et al., 2012, Yucel and Peterson, 2015a, Yucel and Peterson, 2015b) or modifying salt particles’ properties (Hurst et al., 2021, Rama et al., 2013). These approaches are successful as they increase the dissolution speed of sodium from the food matrix to the saliva and the taste receptor cells, increasing the saltiness intensity perceived. Recently, physicochemical design rules for salt particles for salt reduction were proposed, which included: small particle size, low density, low hydrophobicity and optimised particle shape. Salt particles with reduced particle size (<106 µm) and specifically designed spray-dried salt (commercially available as SODA-LO® Salt Microspheres) adopted these design rules and enhanced saltiness perception compared to standard salt particles (106–425 µm) (Hurst et al., 2021).

Food texture impacts food oral processing, which can, in turn, influence the perception of saltiness and the acceptance of products (Jeltema et al., 2015, Lawrence et al., 2012). Modifying food texture to regulate food oral processing is one way that could optimise sodium release in the mouth and enhance saltiness perception. Previously, Tian and Fisk (2012) highlighted that a high proportion of sodium in snacks is consumed without being perceived under regular eating patterns. Therefore, it is hypothesised that increasing the time of the product in the mouth will allow an increased proportion of sodium to be released into saliva and be detected by taste receptor cells. Differences in oral physiology (e.g. saliva flow rate and composition) and individual chewing behaviour can also influence sensory perceptions (Chen, 2009, Lawrence et al., 2012). One way to assess typical chewing behaviour is the JBMB Mouth Behaviour Typing tool**®**, which classifies individuals into four groups (chewers, crunchers, smooshers and suckers) depending on individual preferences for the way one manipulates their foods (Jeltema et al., 2015). Individual mouth behaviour type has been suggested to influence food choice and preferences (Jeltema et al., 2015), and although limited research has been conducted on the influence of mouth behaviour type on saltiness liking and perception, sodium release and saltiness has been shown to be affected by chewing behaviour (Lawrence, Septier, et al., 2012). Texture may also impact saltiness perception through cross-modal interactions, with some studies suggesting that a rough texture can enhance saltiness perception (Biggs et al., 2016, Pflaum et al., 2013, van Rompay and Groothedde, 2019).

Effective salt reduction strategies that allow reduction while maintaining saltiness and consumer acceptance are urgently required, thus a number of salt reduction strategies were investigated. This study firstly aimed to validate physicochemical design rules established previously for salt particles to maximise potential saltiness (small particle size, low density, low hydrophobicity and optimised particle shape) (Hurst et al., 2021). Therefore, prototypes produced using optimised model salts (<106 µm table salt and a spray-dried salt, SODA-LO®) with 30% NaCl salt reduction were assessed within a broader product set of commercially available products and compared to a standard prototype (1.2 g NaCl / 100 g, 106–425 µm salt particle size), using a dual sensory approach. Furthermore, with limited research on salt reduction of potato crisps in literature, this study also investigated: the impact of direct salt removal without any compensatory technique, the effect of changing oral residency time and breakdown speed by altering textural profiles and the impact of ridged texture compared to smooth on consumer acceptance and sensory perception. Additionally, the influence of mouth behaviour on individual perception of potato crisps is explored as variation in oral manipulation and processing is thought to influence product liking. Thus, this study considers the influence of mouth behaviour type on individual perception of salt-reduced potato crisps and commercial competitors.

## 2 Materials and methods

### 2.1 Potato crisp product set

Nine ready salted potato crisp products were used within the study; 3 prototype products were produced using model salt particles topically applied to unsalted potato crisps (Walkers Salt & Shake, PepsiCo, Leicester, UK), and 6 were commercially available products (Table 1).

**Table 1:** Total potato crisp product set. All products outlined were used within the descriptive sensory analysis (n = 9) and for the consumer study, crinkled and low salt commercial products were excluded as they were no longer available (n = 7).

Prototype products				
Prototype reference	Salt particle description	Aimed NaCl content (g/100 g)	NaCl content determined by sodium analysis (g/100 g)^1^	Product description
P1 (STD)	S1: Table salt 106–425 µm (99% NaCl)	1.2	1.18 ± 0.05	Fried sliced potato, Standard NaCl level
P2	M1: Table salt < 106 µm (99% NaCl)	0.84	0.84 ± 0.01	Fried sliced potato, 30% reduced NaCl
P3	M2: SODA-LO® Salt Microspheres Extra Fine salt (93.4% NaCl)	0.84	0.80 ± 0.03	Fried sliced potato, 30% reduced NaCl
Commercial products				
Product reference	Commercial Product name	Declared Back of Pack NaCl content (g/100 g)	NaCl content determined by sodium analysis (g/100 g)	Product description
Low salt	Walkers Hint of Salt	0.9	0.53 ± 0.07	Fried sliced potato, low NaCl
Medium salt	Waitrose Essential Ready Salted	1.15	1.23 ± 0.03	Fried sliced potato, medium NaCl
High salt	Walkers Ready Salted	1.4	1.40 ± 0.10	Fried sliced potato, high NaCl
Baked	Walkers Oven Baked	1.18	1.20 ± 0.10	Baked reconstituted potato flakes, medium NaCl
Crinkled	Walkers Crinkles	1.2	1.12 ± 0.10	Fried crinkle cut potato, medium NaCl
Hand-cooked	ASDA Extra Special Hand cooked crisps	1.2	1.64 ± 0.08	Thicker cut, batch fried potato, medium NaCl

1 Measured salt content presented as mean of 4 replicates ± standard deviation.

#### 2.1.1 Potato crisp prototypes

Three potato crisp prototypes were produced (Table 1). Table salt (99% NaCl) (Sainsbury’s, London, UK), nickel sieves (Fisher, Loughborough, UK) and a coffee grinder (De’Longhi, Treviso, Italy) were used to produce standard salt particles (S1) at a diameter of 106–425 µm, and model salt 1 particles (M1) at a diameter < 106 µm. Model salt 2 (M2) was provided by Tate and Lyle and is commercially available as SODA-LO® Salt Microspheres Extra Fine salt (93.4% NaCl). SODA-LO® is formed by spray-drying a NaCl solution with maltodextrin and has been shown to have a lower density/higher bulk porosity than S1 (Hurst et al., 2021). S1, M1 and M2 were applied to unsalted potato crisps following the method outlined in Rama et al. (2013) using amounts indicated in Table 1 to form the potato crisp prototypes coded as P1 (STD), P2 and P3, respectively. Unsalted potato crisps (100 g) were seasoned by adding the model salt particles to the crisps and tumbling for at least 2 mins in clear polyethylene 300 mm × 250 mm vacuum flat bags (Nisbets, Bristol, UK). Bags were flushed with food-grade nitrogen gas and sealed using a tabletop vacuum packaging machine (Audion Elektro, Derby, UK) to maintain freshness before testing. The weight of the model salt particles (M1 and M2) added to the unsalted potato crisps corresponded to an aimed reduction in the NaCl content of 30% compared to the standard salt level in P1 (1.2 g NaCl / 100 g). A standard NaCl content of 1.2 g/100 g was chosen based on the mode NaCl content in a survey of 30 ready salted fried potato crisp products commercially available in the UK, conducted by the authors (data not shown).

#### 2.1.2 Commercial potato crisp products

Details of commercial products are outlined in Table 1, including NaCl content extracted from the composition tables on the back of pack and NaCl content measured using sodium analysis (method outlined in 2.1.3). To encompass a range of NaCl contents found in the current UK snack market, 3 potato crisp products were included in the product set with low salt (0.9 g/100 g), medium salt (1.15 g/100 g) and high salt (1.4 g/100 g), and are referred to as these descriptors throughout the text. Three additional products were selected with a medium salt content level (1.18–1.20 g/100 g) but differing in cut (crinkle-cut), slice thickness and frying method (thick cut, hand-cooked/batch-fried) and process (baked, reconstituted potato product); these products are referred to as crinkled, hand-cooked and baked in the text and figures. All commercial products were stored within their original packaging at room temperature, away from sunlight, before analysis. The low, medium and high salt potato crisps and all three prototype products (Table 1) are cut straight and were fried using a standard continuous frying method, as was the crinkled potato crisp. All of these products had a crisp thickness of 1.3–1.5 mm. The baked product is made using reconstituted potato flakes to form a dough that is sheeted and oven-baked, providing a product low in fat due to the absence of frying and had a thickness of 1.8 mm. The hand-cooked product is cut thicker than the other products (2 mm) and produced using a ‘kettle’ or batch frying process.

#### 2.1.3 Determination of salt content of products using flame photometry

Analysis of salt content was carried out by One Scientific, Bristol, United Kingdom (UKAS accredited in Food and Food Products). Representative test portions (4 × 50 g each) were sampled from 10 individual crisp packets or 2–3 large crisp packets (depending on pack weight) and incinerated to remove organic material. From the incinerated material, 1–2 g (per replicate) was dissolved in water and diluted to a known volume. Using flame photometry, sodium content of known standards was determined to generate a calibration curve to enable sodium content of product samples to be calculated and converted to salt weight per 100 g of product. Results are presented as mean ± standard deviation of 4 replicates for each product (Table 1).

### 2.2 Descriptive sensory analysis of product set

#### 2.2.1 Panellists

The sensory profiling panel was comprised of 11 panellists (3 Males, 8 Females, 38–68 years old). Panellists were employees of Sensory Dimensions Ltd, Nottingham, UK, and had previously been recruited to the companies trained panel based on their sensory acuity, discriminating ability, motivation and availability. Panellists were highly experienced in sensory descriptive analysis techniques.

#### 2.2.2 Sensory assessment protocol

Due to ongoing COVID-19 restrictions at the time of data collection, all training and feedback sessions took place in panellists' homes using a video calling platform that was easily accessible for all panellists. Panellists took part in five 2-hour training sessions in which panellists familiarised themselves with all nine samples, generated descriptive attributes, and developed the lexicon across appearance, aroma, flavour, texture, and aftertaste (Supplementary Material 1). To understand the sensory characteristics of the whole product set, data across all modalities were collected from the trained panel to avoid ‘dumping’ effects (Abdi, 2002). Three saltiness attributes were included; initial saltiness, overall saltiness during eating and saltiness aftertaste, to provide temporal information on the saltiness profile of the samples. Between sessions 4 and 5, panellists undertook a practice rating assessment to assess panel performance and provide feedback and additional training before the final data collection.

Samples (25 g) were provided to panellists in a 16 oz clear pot with a lid (R&R Packaging, Cramlington, UK) labelled with random 3-digit codes. Samples were stored and served at room temperature. The crisp size was standardised across panellists, i.e. each panellist received similar sizes and amounts of each sample, and small fragments were discarded. A 100-point unstructured line scale was used to rate each attribute, and data was collected using RedJade Sensory Solution Software, 2021 version (RedJade, Martinez, CA, USA). All panellists rated each sample in duplicate over 3 different sessions, tasting 6 samples in each session. The order of samples was randomised across the whole panel. Panellists were instructed to take a 5-minute break between each sample evaluation and cleansed their palate with unsalted crackers and tap water from panellists homes to reduce carry-over effects. Panellists were instructed to perform evaluations in the same place each time with no distractions.

### 2.3 Consumer acceptance of product set

Quantitative consumer acceptance testing was performed on a reduced product set (Table 1) due to 2 products (low salt and crinkled potato crisps) being delisted during the study.

#### 2.3.1 Consumers

93 consumers (45 Males, 48 Females) were recruited from the Sensory Dimensions Ltd consumer volunteer database via a screening questionnaire. Participants were recruited using the inclusion criteria; 18–65 years old with no food-related allergies, in good health with no salt-related health issues (such as hypertension, cardiovascular disease, kidney disease), no taste impairments, must be a regular crisp user (at least once a month) and must accept ready salted potato crisps. Information on demographics’ (gender, age, and ethnicity) and frequency of snack product usage were captured using a self-report questionnaire.

#### 2.3.2 Consumer assessment protocol

Consumers were invited to central location test facilities in the UK, and sample assessment took place within sensory booths (ISO 8589:2007). Samples were labelled using random 3-digit codes, and consumers assessed the 7 samples within one session following a sequential monadic presentation with a randomised order across consumers. Consumers were provided with 10 g of each sample to assess overall liking, saltiness liking and texture liking using 9-point hedonic category scales where 1 represented “dislike extremely” and 9 represented “like extremely”. For saltiness, they were provided with a 5-point Just-About-Right (JAR) scale where 1 represented “not at all salty enough”, 3 represented “Just-about-Right” and 5 represented “far too salty”. At the end of the final tasting session, consumers completed an online questionnaire about the preferred way they like to manipulate different types of foods in their mouth (JBMB Typing Tool**®**) in order to classify their mouth behaviour preferences (Jeltema et al., 2015). Based on their responses, consumers were grouped into four different mouth behaviour types: smooshers, crunchers, suckers, and chewers.

### 2.4 Ethical statement

all products were commercially purchased or produced from commercially available food-grade materials in food-grade environments using no novel ingredients. Participants provided their informed consent before taking part in the study. Testing procedures followed sensory, marketing and consumer research protocols including; ISO 11136, IFST Professional Sensory Science Group and Market Research Society guidelines (British Standards Institute, 2017, IFST Sensory Science Group, 2020, Market Research Society, 2022).

### 2.5 Data analysis

For all data analysis, an α risk of 0.05 was set as the level of significance and, unless otherwise stated, was conducted using XLSTAT (19.01, Addinsoft, New York, USA).

#### 2.5.1 Descriptive sensory analysis

To determine if differences existed between products for each attribute assessed by panellists, a two-way fixed model ANOVA (product, panellist) with interaction was performed, followed by Tukey’s HSD post-hoc test. Panel performance was evaluated using SenPAQ (version 5.01, Qi Statistics, Berkshire, UK).

#### 2.5.2 Consumer acceptance analysis

To determine if differences existed between products for overall liking and specific attribute liking, a two-way mixed model ANOVA (product, consumer), with the consumer as a random effect, was performed, followed by Tukey’s HSD post-hoc test. Agglomerative hierarchical cluster analysis using a dissimilarity matrix with Euclidean distance and Ward’s method in the agglomeration was applied to classify consumers by patterns in overall liking. ANOVA was performed on the resulting clusters to determine if significant differences in liking existed between products within each cluster group.

Overall liking scores and saltiness JAR scores were used for penalty analysis following the steps outlined using the sensory analysis tool on XLSTAT (19.01, Addinsoft, New York, USA) and the procedure in Schraidt (2009). This analysis was performed for the whole consumer group (n = 93) and each liking cluster group and mouth behaviour group. Chi-square and Fisher’s exact tests were used to explore the differences in demographic, product usage and behavioural characteristics among cluster membership and mouth behaviour types.

An internal preference map was constructed using a PCA bi-plot of the multivariate space of the products, with mean overall liking scores of cluster groups and sensory attributes as supplementary variables to visually display and explore drivers of liking and disliking for each cluster.

## 3 Results

### 3.1 Salt content of the product set

Salt content was assessed to check the accuracy of the declared salt content found on the back of pack (BoP) for commercial products and to ensure prototype samples were within the desired salt content range to exhibit a 30% salt reduction between standard salt level (P1, STD) and reduced salt prototypes (P2, P3). Measured NaCl content was within range for BoP reported content for most products (Table 1), with the exception of the low salt product (BoP: 0.9 g/100 g, measured: 0.53 g/100 g) and the hand-cooked product (BoP: 1.2 g/100 g, measured: 1.64 g/100 g). Prototype products ultimately met the aimed salt levels to achieve two 30% salt reduced prototypes (P2 and P3) compared to the standard prototype (P1). The declared BoP and aimed salt content values are referred to through the text of this paper for ease, where the measured salt content is similar to BoP. Any implications of out of specification products are discussed where appropriate in the text.

### 3.2 Overall descriptive sensory profiles of the product set

**Fig. 1:**
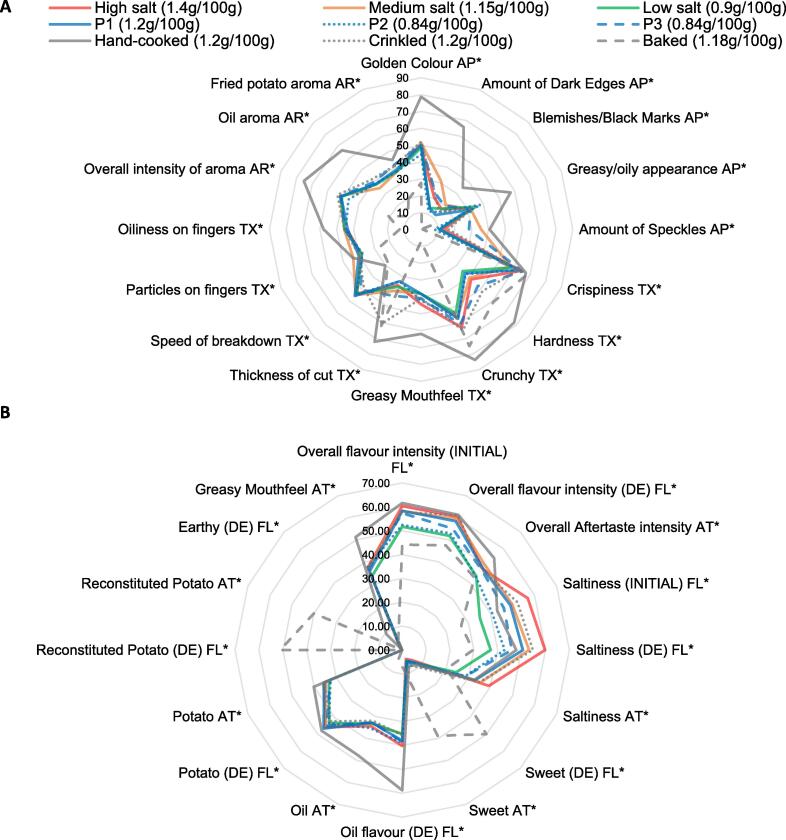
Spider plots of mean attribute ratings of 9 potato crisp products^1^ assessed by 10 panellists. Graph A presents aroma (AR), appearance (AP) and texture (TX) attributes. Graph B presents flavour (FL) and aftertaste (AT) attributes. Asterisks detail that a significant difference exists between products for that attribute (p < 0.05).

The evaluation of panel performance indicated that one panellist performed poorly relative to the rest of the panel in discrimination ability, repeatability, and consistency and was removed from the data set. The mean scores for all 32 attributes measured across the product set are presented in spider plots in Fig. 1a-b. ANOVA results indicated that for all attributes, significant differences were observed (p < 0.0001). For clarity, post-hoc groupings are not included in Fig. 1 spider plots. This information can be found in Supplementary Material 2a-d along with the mean scores and standard deviation of each attribute. Fig. 1a-b confirms that the three prototype products (P1 STD, P2, P3) have similar sensory profiles, with P3 being significantly higher in some appearance attributes (amount of speckles and amount of dark edges) and texture attributes (hardness and thickness of cut) (p < 0.05). Fig. 1a-b also indicates that the hand-cooked and baked products have the most contrasting sensory profiles from the product set, as expected, due to different cooking processes and crisp thickness. The remaining products exhibit similar sensory profiles across attributes with the most noticeable differences in flavour intensity attributes (initial and during eating) and the three saltiness attributes (initial, during eating and aftertaste). Fig. 1a-b show that the hand-cooked product had significantly higher values in: all aroma and appearance attributes, most texture and mouthfeel attributes (greasiness, hardness, crunchiness) and some flavour and aftertaste attributes (earthy and oil) (Supplementary Material 2a-d). The baked product, on the other hand, was significantly lower in all aroma attributes, most appearance attributes and all oil associated attributes (flavour, texture, mouthfeel) (p < 0.05) (Supplementary material 2a-d). Baked also had significantly higher sweetness attributes and attributes associated with reconstituted potato than other products (p < 0.05).

#### 3.2.1 Saltiness profiles of the product set

**Fig. 2:**
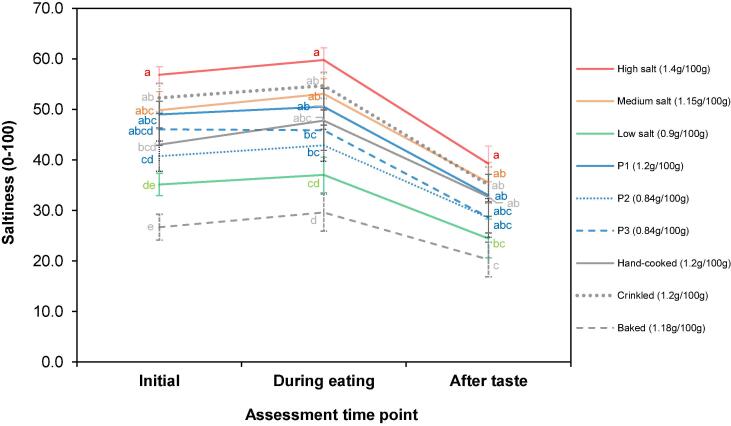
Saltiness intensity of 9 potato crisp products^1^ assessed by panellists (n = 10) at 3 different assessment points via sensory descriptive analysis. Different letters within each assessment time point show significant differences (p < 0.05). Error bars represent standard error.

For each saltiness attribute (initial, during eating and aftertaste), significant differences were identified between the product set (p < 0.0001) (Fig. 1b). Fig. 2 displays the mean scores and post-hoc groupings for each saltiness rating across the product set. Most products slightly increase in saltiness from the initial rating to the rating during eating; saltiness intensity then decreases when assessed as an aftertaste (Fig. 2). Products were ranked predominantly in the same order of saltiness intensity for each saltiness attribute, with the high salt product (1.4 g NaCl / 100 g) being the saltiest and the least salty being the baked product (1.18 g NaCl / 100 g). No significant differences were observed between prototypes for all three saltiness attributes measured (p > 0.05). In addition, when comparing the salt-reduced prototypes (P2, P3) to a commercially relevant competitor at a medium salt level (1.15 g NaCl / 100 g), there were no significant differences between products for each saltiness attribute (p > 0.05) (Fig. 2). Moreover, there was no significant difference in saltiness during eating between P3 and the high salt product (1.4 g NaCl / 100 g) or between P2, P3 and the high salt product for saltiness aftertaste. However, there was no significant difference between P2, P3 and the low salt product (0.9 g NaCl / 100 g).

Crinkled and hand-cooked products (1.2 g NaCl / 100 g) were not significantly different to the standard prototype product (P1: 1.2 g NaCl / 100 g) nor the medium potato crisp product (1.15 g NaCl / 100 g) (p > 0.05). The baked product (1.18 g NaCl / 100 g) compared to the rest of the product set was significantly different across all saltiness attributes, except for the low salt product (0.9 g NaCl / 100 g) (Fig. 2). No significant differences were found between the high salt (1.4 g NaCl / 100 g) and medium salt potato crisps (1.15 g NaCl / 100 g and P1 1.2 g NaCl / 100 g) for all three saltiness attributes. However, there was a significant difference between the low salt and high salt product (p < 0.05) for each saltiness attribute.

### 3.3 Consumer acceptance of the product set

#### 3.3.1 Consumer panel general demographics and crisp consumption habits

Information on demographics, mouth behaviour type and product usage characteristics can be found in Table 2 for the total consumer group. The majority of the consumers identified as white British (90%), and there was an even spread of consumers across age ranges (18–24 years: 14%, 25–34 years: 12%, 35–44 years: 17%, 45–54 years: 27% and 55–65 years: 30%). The consumer group tested were frequent crisp consumers; all consumed crisps at least once a month, with 82% of consumers stating that they ate crisps more than two times a week.

**Table 2:** Demographic, mouth behaviour type and potato crisp consumption habits for the total consumer group and each cluster.

	Frequency response (%)			
	Total consumers (n = 93)	Cluster 1 n = 21	Cluster 2 n = 43	Cluster 3 n = 29
**Product liking characteristics**		Baked product dislikers	Liked Medium-salt product least	Hand-cooked product dislikers
**Gender**				
Female	52%	38%	56%	55%
Male	48%	62%	44%	45%
**Age**				
18–24	14%	10%	12%	21%
25–34	12%	5%	14%	14%
35–44	17%	5%	16%	28%
45–54	27%	38%	26%	21%
55–64	30%	43%	33%	17%
**Mouth behaviour**				
Chewer	22%	14%	19%	31%
Cruncher	35%	29%	40%	34%
Smoosher	10%	14%	5%	14%
Sucker	23%	24%	26%	17%
Unclassified	11%	19%	12%	3%
**Consumption habits (any flavour)**				
Less than once a week	7%	5%	12%	3%
Once a week	10%	19%	7%	7%
2–4 times a week	57%	52%	51%	69%
Once a day or more	25%	24%	30%	21%
**Consumption habits (ready salted)**				
Less than once a week	21%	33%	21%	14%
Once a week	29%	14%	30%	38%
2–4 times a week	44%	43%	42%	48%
Once a day or more	4%	10%	7%	0%
**Normal commercial crisp consumption**				
Walkers ready salted	83%	95%	67%	97%
Walkers Hint of Salt	14%	10%	7%	28%
Supermarket own-brand ready salted crisps	43%	48%	33%	55%
Walkers oven baked sea salt	44%	33%	40%	59%
Kettle original sea salt	45%	67%	44%	31%
Pringles original salted	70%	57%	72%	76%
Smiths original crinkle cut salted	11%	14%	7%	14%
McCoys Ridge Cut salted	49%	71%	35%	5%
Originally Smiths Walkers salt and shake	17%	24%	14%	17%
Tyrells Lightly salted	26%	33%	30%	14%
Walkers crinkles simply salted	30%	14%	28%	45%
Popchips sea salted	6%	5%	12%	0%
Pom Bear Original	29%	19%	26%	41%

#### 3.3.2 Attribute liking ratings, saltiness just-about-right scores and penalty analysis of the total consumer group

**Fig. 3:**
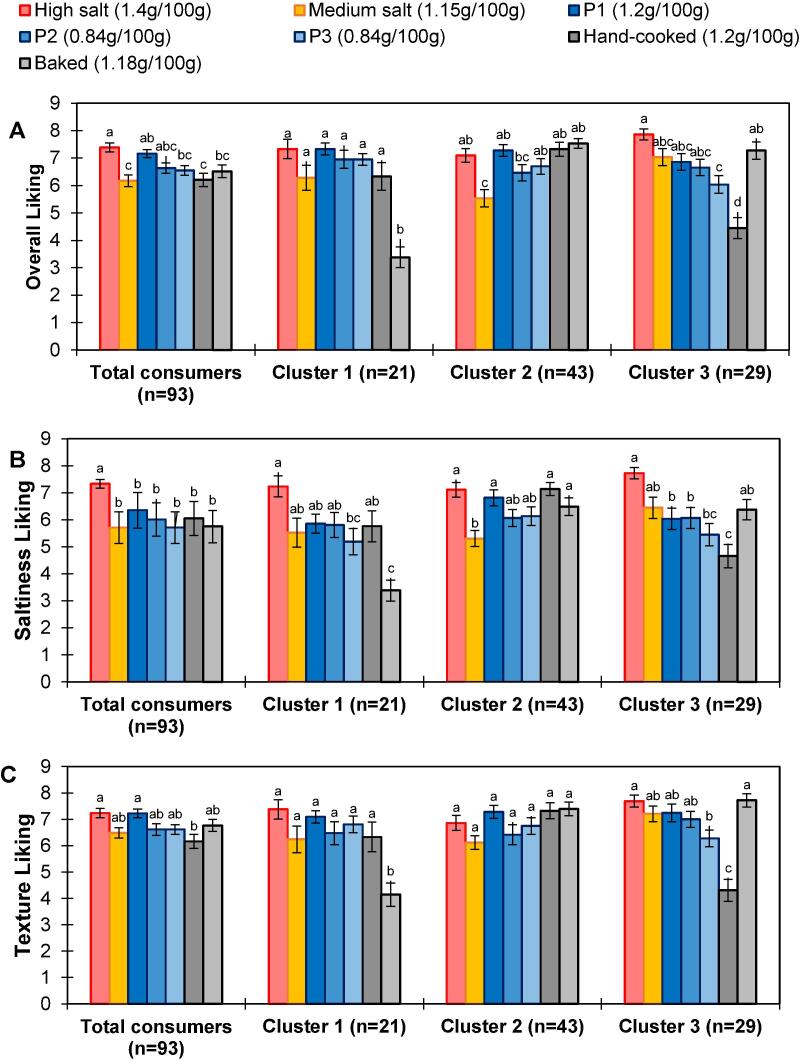
Liking scores for each liking attribute per product^1^ calculated for total consumer group (n = 93) and for each cluster group. Different letters within cluster group or total consumer group determine a significant difference (p < 0.05). Error bars represent standard error.

Fig. 3a-c describes the mean attribute liking scores across the whole consumer group (n = 93) for each product, along with post-hoc groupings. ANOVA revealed significant differences between the product set across all liking attributes (p < 0.0001), yet, there was no significant differences in overall liking, saltiness liking and texture liking between the standard prototype (1.2 g NaCl / 100 g) and either of the model salt prototypes with 30% less NaCl (P2, P3: 0.84 g NaCl / 100 g) (Fig. 3a-c). The high salt product (1.4 g NaCl / 100 g salt) had the highest overall liking (7.4 ± 1.5), which was significantly higher than all the products except P1 (STD) and P2 (Fig. 3a). The hand-cooked product was, on average, the least liked (6.2 ± 2.3), significantly less so than the high salt product and P1 (STD) (Fig. 3a). Similar to overall liking scores, the high salt product had the highest mean saltiness liking score (7.4 ± 1.5), which was significantly higher than all other products (p < 0.05). No significant differences were observed between all three prototype products and the remaining products (p > 0.05) (Fig. 3b). Fig. 3c shows the most liked products for texture were the high salt product and P1 (STD), whose values were significantly higher than the hand-cooked (p < 0.05) but not significantly different to all other products, including the salt-reduced prototypes (P2, P3) (p > 0.05).

**Fig. 4:**
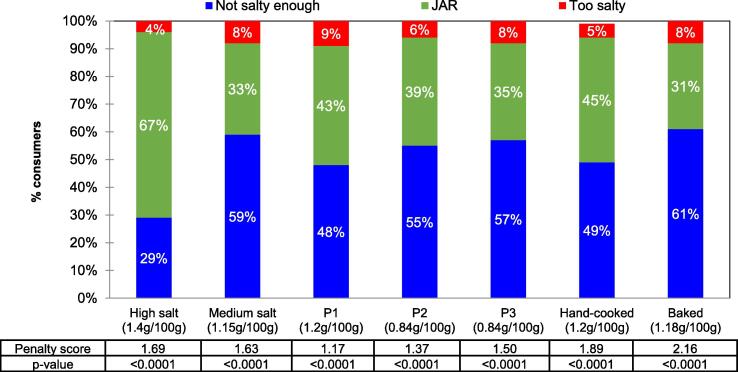
Just-about-right (JAR) responses^2^ for each product^1^ of the whole consumer base (n = 93) shown as a percentage. The table underneath the graph details the penalty score^3^ and p-value^4^ from the penalty analysis.

Fig. 4 shows the Just-About-Right (JAR) responses for each product and the penalty score, which is the reduction in mean liking between JAR respondents and non-JAR respondents, calculated by penalty analysis. All products showed unbalanced JAR profiles with few consumers responding ‘too salty’ (4–9%). With the exception of the high salt product (1.4 g NaCl /100 g), other products had a high percentage of ‘not salty enough’ responses (48–61%). The product with the highest salt level had the greatest proportion of JAR responses (67%), whilst the baked product had the lowest (31%). The results from the penalty analysis indicated that all products not being salty enough or too salty is associated with a significant drop in overall liking of between 1.17 and 2.16 on a 9-point liking scale (table in Fig. 4). The prototypes had similar JAR profiles (Fig. 4). Although there was a slightly lower percentage of consumers who rated P1 as not salty enough (48%) compared to P2 and P3 (55% and 57%, respectively), these were similar to the medium salt product (59%). When observing the penalty score (table in Fig. 4), P3 decreased by (1.50) while P1 (STD) and P2 only decreased by 1.17 and 1.37, respectively.

#### 3.3.3 Consumer cluster groups based on overall liking patterns

To identify groups of consumers differing in overall liking patterns, a segmentation of the consumer group was performed by Agglomerative Hierarchical Clustering, which identified three groups (Supplementary Material 3). ANOVA revealed a significant interaction effect between the product and cluster group for all three liking attributes (p < 0.001), indicating that attribute liking varied depending on the consumer cluster. Fig. 3a-c show mean attribute liking scores across each cluster group. Significant differences were found between the product set for overall, saltiness and texture liking within each cluster group (p < 0.001) (Fig. 3a-c). Of these differences observed, no significant differences between the three prototype products were found within the three cluster groups for all three liking attributes (p > 0.05). Cluster 1 (23%) disliked the baked product (Fig. 3a-c), with 100% of cluster 1 also rating baked as ‘not salty enough’ (Supplementary Material 4). Cluster 2 (46%) liked the commercial medium salt product the least, and cluster 3 (31%) disliked hand-cooked (Fig. 3a-c), which also had the highest number of consumers (71%) rating it as ‘not salty enough’ (Supplementary Material 4). The high salt product was liked highly across all three cluster groups (Fig. 3a-c).

Table 2 presents the demographic, mouth behaviour type and product usage characteristics for each cluster group. Chi-square and fisher test results (not presented) confirmed that clusters were independent of mouth behaviour type, gender and crisp consumption frequency. However, cluster 1 had fewer consumers in the age category 35–44 years while cluster 3 had fewer consumers in the age range of 55–64 years. Unsurprisingly, cluster 1 (lower liking for the baked) has a lower percentage of consumers (33%) who stated that they normally consume a baked variety of crisps than the other two clusters (40–52%). Additionally, cluster 3 (lower liking for hand-cooked) had the lowest proportion of members who stated that they usually consume hand-cooked crisp varieties (Table 2).

#### 3.3.4 Internal preference mapping

**Fig. 5:**
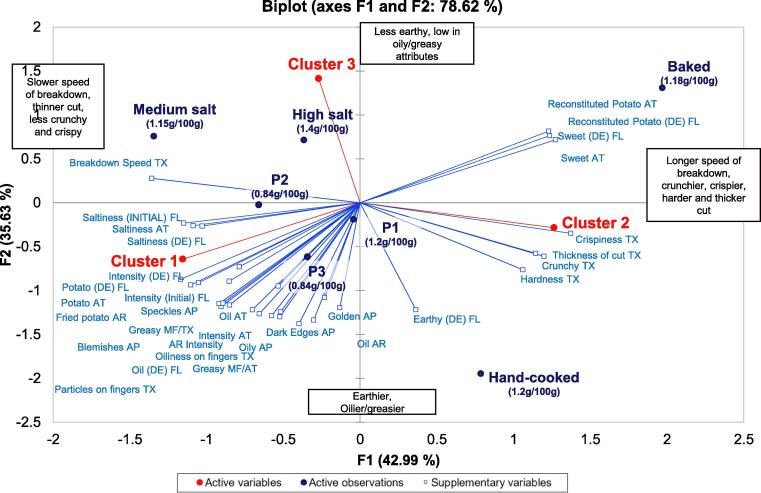
Internal preference map of mean overall liking data for each cluster with mean sensory attribute scores as supplementary variables. Each cluster is represented in red, the active observations in dark blue are the products^1^ and the supplementary variables in light blue are the sensory attributes. (For interpretation of the references to colour in this figure legend, the reader is referred to the web version of this article.)

Internal preference mapping (Fig. 5) was used to visualise overall preferences of the consumer clusters against the product sensory profiles. Two dimensions accounted for 78.62% of the total variance (42.99% and 35.63% for F1 and F2, respectively), with both axes separating the three cluster groups within the multivariate space. F1 separated consumer cluster 1 (23%) and cluster 2 (46%) as F1 was strongly negatively correlated with cluster 1 overall liking (-0.757) and strongly positively correlated with cluster 2 (0.828). F2 was strongly positively correlated with cluster 3 (31%) (0.926), separating this cluster from the other two. Overall liking of cluster 1 was significantly positively correlated with initial flavour (0.806), initial saltiness intensity (0.863), saltiness during eating (0.819), potato flavour (0.944), greasy mouthfeel (0.759), oiliness on fingers (0.815), saltiness aftertaste (0.904) and breakdown speed (0.772) while being significantly negatively correlated with sweetness (-0.960), reconstituted potato flavour (-0.952). Overall liking of cluster 2 was significantly positively correlated to crispiness (0.886). Cluster 3 overall liking was significantly negatively correlated with oil aroma (-0.767), golden appearance (-0.767), dark edges (-0.797), and earthy flavour (-0.658).

#### 3.3.5 Mouth behaviour typing using JBMB typing tool®

Using the JBMB typing tool® (Jeltema et al., 2015), 83 consumers were grouped according to mouth behaviour type (Table 2). Ten consumers could not be classified due to technical issues completing the surveys and were excluded from the subsequent analysis based on mouth behaviour type. After removing those unclassified, the most predominant mouth behaviour type was the crunchers (33%), followed by suckers (24%), chewers (20%) and smooshers (11%). Demographic and product usage characteristics of each mouth behaviour group is displayed in Supplementary Material 5. Chi-square and Fisher exact tests (results not presented) determined that mouth behaviour type was independent of gender, age and frequency of crisps consumption (any flavour and ready salted). ANOVA revealed no interaction effect between product and mouth behaviour group for overall, saltiness and texture liking attributes (p = 0.41–0.89), suggesting that liking was not influenced by mouth behaviour type (Supplementary Material 6). There was, however, a main effect of mouth behaviour type on texture liking (p = 0.01). The subsequent post-hoc test identified that chewers and crunchers gave significantly higher scores for texture liking than suckers (p < 0.05) (Supplementary Material 7).

## 4 Discussion

This study aimed to validate the potential for NaCl salt reduction by leveraging physicochemical design rules set out to create optimised model salt particles (Hurst et al., 2021). The previous study recommended a series of design suggestions for salt particles: small particle size, low density, and low hydrophobicity with a particle shape associated with optimal flow properties, which are applicable to the model salts selected for this current study. The model salts selected, reduced particle size (<106 µm) and a spray-dried salt with maltodextrin (commercially available as SODA-LO®), were previously shown to enhance sodium dissolution, saltiness perception, transfer efficiency and adhesion when applied to peanuts. SODA-LO® and < 106 µm table salt have similar mean particle sizes; however, the morphology of SODA-LO® shows a higher surface area due to internal voids which enhances dissolution rate. Prototype products made using these salts at a 30% salt reduced level were assessed and compared to a broader product set, including commercially available products, using a dual sensory and consumer assessment approach. Since this research uses mainly topically applied products, our findings may also apply to other topically applied snacks such as peanuts and popcorn.

### 4.1 Descriptive sensory analysis and saltiness profiles of the product set

Application of model salt particles to potato crisps that followed physicochemical design rules (Hurst et al., 2021) enabled a 30% reduction in NaCl content since no significant difference in saltiness intensity was found across 3-time points: initial, during eating and aftertaste (p > 0.05) when compared to the standard prototype (P1: 1.2 g NaCl / 100 g) and a commercially available medium salt product (1.15 g NaCl / 100 g). Additionally, P3 was not significantly different in initial saltiness nor saltiness aftertaste compared to the highest salt content product (1.4 g NaCl / 100 g, p > 0.05), suggesting that the model salt (SODA-LO®) used to prepare the salt-reduced prototype (P3) could be successful in reducing NaCl content, even further, up to 40%. Nevertheless, saltiness during eating was significantly different between P3 and the high salt product. No significant differences between prototype products were seen in most of the other attributes assessed (Supplementary Material 2a-d). The observed differences (some appearance attributes, hardness, and cut thickness) are probably due to the batch to batch variation of the unsalted crisps commercially purchased, despite randomisation of batch codes when generating the crisp prototypes. These slight differences are unlikely to impact saltiness perception. Thus, any changes in saltiness perception across the 3 prototypes can be attributed to the design of salt particles. Overall, we have validated that the physicochemical design rules (Hurst et al., 2021) can reduce NaCl by 30% without significantly affecting saltiness perception.

Consistent with other research (Drake et al., 2011, Jaenke et al., 2017, La Croix et al., 2015, Levings et al., 2014, Mueller et al., 2016), our results suggest that limited salt reduction could be possible by direct NaCl removal without perceivable impact. Results indicated no significant difference in saltiness between the standard prototype (1.2 g NaCl / 100 g) and the high salt level product (1.4 g NaCl / 100 g), a reduction of approximately 15% based on measured NaCl content (Table 1). These products were used to determine potential level of salt reduction since the base potato crisp was supplied by the same manufacturer (see section 2.1).

Previous studies have suggested that a rough texture, i.e. ridges or crinkles compared to a smooth texture, could enhance saltiness perception (Biggs et al., 2016, Pflaum et al., 2013, van Rompay and Groothedde, 2019). However, in the current study, no significant difference was seen in saltiness intensity between standard flat potato crisp and the crinkled potato crisp at similar NaCl contents (Table 1), supporting evidence from Kongstad and Giacalone (2020) that differences in the cut of potato do not influence the perception of saltiness.

The baked product is the only product in the set that contained NaCl salt in both the matrix and outside the product. The addition of salt within the potato base dough contributes to the desired flavour and texture of the final product during baking. Despite the baked product having the same NaCl salt content as medium salt level products (Table 1), it was significantly the least salty, suggesting that a proportion of salt contained in the baked product goes unperceived as a result of being ‘trapped’ within the matrix, thus, reducing its availability to interact with the taste receptor cells. Limited research has been published on the impact of salt removal and salt reduction strategies within the matrix of baked and reconstructed snacks, e.g. thermomechanically extruded products. It would be a valuable area for further investigation, especially since expanded snacks often require much higher NaCl contents to develop the desired texture (van der Sman & Broeze, 2013).

Previously, Tian and Fisk (2012) highlighted that a proportion of sodium is consumed without being perceived under regular eating patterns of potato crisps. A thick cut hand-cooked product and a reconstituted product were included in the study to test whether texture and oral breakdown speed increase the proportion of perceived sodium. Despite the baked and hand-cooked products reducing the breakdown speed (hypothesised to optimise the release of sodium for detection by the taste receptor cells), their saltiness profiles were not enhanced significantly (p > 0.05). Since salt was topically applied, any available salt will dissolve in saliva within the first 10–20 s (Hurst et al., 2021) and therefore, break down speed or the subsequent length of time in mouth did not affect perception as any available NaCl would probably be dissolved rapidly at the beginning of consumption. However, chewing behaviour is more likely to affect perception when salt is incorporated into the food matrix and has a slower release of sodium ions from the matrix (Lawrence, Septier, et al., 2012), so this could be an exciting area to investigate to optimise salt release in the mouth. Surprisingly, the hand-cooked product did not have the highest perceived saltiness even though Table 1 shows that it has the highest measured NaCl content (1.64 g/100 g), despite declared BoP content being 1.2 g/100 g. This could be attributed to a larger particle size (not measured in this study) or the higher level of greasiness suppressing saltiness perception (Supplementary Material 2b), due to oil acting as a barrier for tastant delivery to taste receptor cells (Metcalf & Vickers, 2002). On the other hand, this disparity may also be due to the high variability in NaCl content between product batches.

### 4.2 Consumer acceptance test of the product set

#### 4.2.1 Attribute liking ratings, saltiness just-about-right scores and penalty analysis of the total consumer group

Results indicated that consumers liked the high salt product (1.4 g NaCl / 100 g) the most. This product had an overall liking score that was significantly higher than P3, hand-cooked, baked and medium salt products (Fig. 3a). Similarly, the high salt product had significantly higher saltiness liking than all other products, with the highest percentage of saltiness JAR responses (67%) (Fig. 4). In contrast, all other products have a much greater percentage of consumers reporting that the product is ‘not salty enough' (Fig. 4). The results from the penalty analysis in Fig. 4 also revealed that liking was reduced significantly across all products when the product was not considered salty enough or too salty by the consumer. Although all products were at least ‘slightly liked’ (Fig. 3), our findings highlight the difficulty in reducing salt whilst retaining consumer acceptance, given the common preference towards higher salted potato crisps. Undoubtedly, food manufacturers cannot increase the salt content of products to obtain more balanced JAR profiles; however, these results highlight the importance of saltiness as a driver of liking.

Consistent with the saltiness profile results (Fig. 2), there were no significant differences between the three prototype products (Fig. 3a-c) for all three liking attributes. Therefore, salt content can be reduced by up to 30% while maintaining saltiness liking and overall liking when using salt particles that adopt physicochemical design rules. Furthermore, saltiness ratings (as discussed in section 4.1) suggested that a 15% (based on measured salt content) salt reduction via direct salt removal could be possible without significantly affecting saltiness perception. Overall liking scores confirm that this level of reduction through direct salt removal may be possible without impacting consumer acceptance since there was no significant difference between the high salt product (1.4 g/100 g) and standard prototype (1.2 g/100 g).

#### 4.2.2 Consumer cluster groups based on overall liking patterns

Consumer segmentation groups were driven by preference for texture profiles which were altered by processing methods (e.g. batch fried product and reconstituted baked product) (Fig. 3 and Fig. 5). Cluster 1 (23%) was characterised by disliking the baked product; cluster 2 (46%) was characterised by liking the medium salt commercial product the least; cluster 3 (31%) was characterised by disliking the thick-cut, hand-cooked product. Despite this, the most liked product across all cluster groups remained the highest salt product, further evidencing that most consumers seek a high salt level in their snacks. More research is required into altering saltiness preferences, for example, the role of early exposure to salt, to avoid damaging health impacts due to high NaCl salt consumption (DeSimone et al., 2013).

Drivers of liking are typically determined to understand consumer preferences towards products and the desirable sensory attributes required for high consumer acceptance. Drivers of liking were determined for each cluster group using the internal preference map in Fig. 5, and correlations found between cluster liking and sensory attributes (presented in section 3.2.2). Cluster 1 (23%) prefer products with a high potato flavour intensity, high saltiness, greasy mouthfeel and quick speed of breakdown, while cluster 2 (46%) drivers of liking include crispiness and slow breakdown speed. Drivers of liking for cluster 3 (31%) include low oil aroma, low earthiness and light in colour. The attributes identified as drivers of liking for each cluster group reflect each clusters’ most preferred products. Surprisingly, mouth behaviour type was not associated with cluster groups despite the differences in cluster groups being driven by texture, which is the primary determinant of mouth behaviour type (Jeltema et al., 2015).

#### 4.2.3 Mouth behaviour typing using JBMB typing tool®

The JBMB typing tool® was used to characterise consumers by their preferred mouth behaviours. The proportion of consumers characterised as suckers in this study (24%) was surprisingly higher than previously reported (8%) (Jeltema et al., 2015). Nevertheless, differences in the spread of mouth behaviour groups have been reported between countries, with the proportion in this study being closer to that found in a UK cohort (15%) (Licker, 2019). The relatively high proportion of suckers in this study is also surprising since the inclusion criteria was frequent crisp users (consuming crisps at least once a fortnight). It might be expected that a group of high-frequency crisp consumers would have a relatively lower proportion of suckers since suckers prefer foods that they can suck on until they dissolve like hard candies, whereas crisps may appeal more to crunchers as crisps are typically crispy and crunchy products. Therefore, the prevalence of suckers in our study suggests that mouth behaviour preference does not ultimately define an individual’s consumption habits or that despite a hard and crunchy texture, individuals can still use their preferred mouth behaviours to manipulate the texture of the product. Although it was found that the mouth behaviour group did not impact overall and saltiness liking of the different products, there was a main effect of mouth behaviour group on texture liking (Supplementary Material 7). Suckers texture liking ratings were significantly lower across the whole product set compared to chewers and crunchers (p < 0.05). This is expected since crunchers are characterised by their preference towards food textures that they can crunch like crunchy granola, crispy vegetables, and crunchy cookies and chewers have a preference towards food that they can chew on (Jeltema et al., 2015).

### 4.3 Limitations of study

This study provides preliminary data investigating the effect of mouth behaviour type on consumer’s crisp preferences; however, to fully explore the impact of mouth behaviour on perception and preference, a larger consumer group and a wider range of product textures should be considered. Increasing the number of consumers would also enable more robust segmentation to explore key differences between cluster group characteristics. Consumer choice is influenced by factors outside of a product's inherent sensory characteristics, including branding, packaging design, and nutritional information. For example, in a previous study, when consumers were informed of reduced salt content of potato crisps on the packaging, consumers preferred the reference product over the salt reduced crisps; however, when tested blind, this was not the case. Therefore, there is a strong labelling effect on potato crisp product liking and saltiness perception (Kongstad & Giacalone, 2020). Products within this study were assessed unbranded to enable the study’s primary aim of determining the capability of the model salts to retain the perception of saltiness whilst reducing salt content. Nevertheless, the impact of these extrinsic product factors on preference and purchasing intent should not be disregarded when adopting salt reduction strategies by food manufacturers.

## 5 Conclusions

This study was designed to evaluate the application of model NaCl salt particles, which adopt the salt reduction design rules in Hurst et al. (2021), to a common salty snack product: potato crisps. Potato crisp prototypes produced using model salts (<106 µm table salt and a spray-dried salt, SODA-LO®) with 30% salt reduction were assessed within a broader product set of commercially available products to understand sensory and consumer acceptance performance within the market context. Overall, this study successfully demonstrated a route to 30% salt reduction in a topical application, without loss of perceived saltiness intensity or impact on consumer liking, therefore validating the previously proposed physicochemical design rules for the formulation of model salt particles. Broader results suggest that salt reduction through direct salt removal (without compensating for reduction)may be successful, but only by 15%, providing further evidence that specifically designed salt particles for salt reduction are required. Despite previous suggestions that roughness may enhance saltiness perception, crinkled potato crisps did not enhance the saltiness through cross model interactions. This study also investigated the key drivers of liking and segmented consumers based on their overall liking patterns and mouth behaviour type. While varying textural profiles, caused by different processing techniques segmented consumers by overall liking, mouth behaviour groups did not differ in their preferences across the product set. Despite variations in oral breakdown speed across some products, saltiness perception was not enhanced by the increased time in mouth for salt dissolution. The presented findings will be of interest to both the food industry and academic researchers. Specifically, it will be relevant to those actively looking to reduce salt in foods containing topically applied seasonings and salt without compromising the perception of saltiness and consumer liking.

## Funding statement

This work was supported by the BBSRC [grant number BB/N021126/1]; Innovate UK [grant number 104430]; and the University of Nottingham.

### CRediT authorship contribution statement

**Katherine E. Hurst:** Conceptualization, Methodology, Formal analysis, Investigation, Writing – original draft, Visualization. **Louise Hewson:** Conceptualization, Methodology, Writing – review & editing, Supervision. **Ian D. Fisk:** Conceptualization, Writing – review & editing, Supervision, Funding acquisition.

## Declaration of Competing Interest

The authors declare that they have no known competing financial interests or personal relationships that could have appeared to influence the work reported in this paper.

## Footnotes

1 Potato crisp products include prototypes (P1, P2, P3) with the rest being commercial products detailed in the text. Prototype 1 (P1) was made using 1.2 g of 106–425 µm NaCl salt per 100 g of unsalted potato crisps. P2 and P3 are 30% reduced NaCl salt products when compared to P1 and were made using < 106 µm NaCl salt or SODA-LO® salt microspheres respectively.

2 Responses “not quite salty enough” and “not at all salty enough” were grouped into the “not salty enough” category and responses “too salty” and “far too salty” were grouped into the “too salty” category.

3 Decrease in overall liking between respondents who scored products as JAR and those who scored samples at the other two levels (not salty enough and too salty).

4 p-value determined by the comparison test of the overall liking mean for JAR respondents and those who scored samples at the other two levels (not salty enough and too salty).
