# Supplementary material for: Sensory perception and consumer acceptance of commercial and salt-reduced potato crisps formulated using salt reduction design rules
Source: Food Res Int. 2022 May;155:111022. doi: 10.1016/j.foodres.2022.111022 (PMC9022087; doi:10.1016/j.foodres.2022.111022)
Supplement: Supplementary data 1 [file mmc1.docx]

**Supplementary Material**

**Sensory perception and consumer acceptance of commercial and salt-reduced potato crisps formulated using salt reduction design rules**

Katherine E. HURST^a^, Louise HEWSON^a^, Ian D. FISK^ab*^

^a^ Division of Food, Nutrition and Dietetics, University of Nottingham, Sutton Bonington Campus, Loughborough LE12 5RD, United Kingdom.

^b^ The University of Adelaide, North Terrace, Adelaide, South Australia, Australia

Katherine E. Hurst: <https://orcid.org/0000-0002-1268-2550>

Louise Hewson: <https://orcid.org/0000-0001-6630-7089>

Ian D. Fisk <https://orcid.org/0000-0001-8448-3123>

Corresponding author: [**Ian.fisk@nottingham.ac.uk*](mailto:*Ian.fisk@nottingham.ac.uk)

| Supplementary material 1. Attributes and definitions used in descriptive analysis of 9 crisp products. | |
| --- | --- |
| Sensory attributes | Definition |
| *Aroma* |  |
| Overall intensity | The overall intensity of aroma. |
| Oil | The intensity of aroma associated with fresh hot cooking oil. |
| Potato | The intensity of aroma associated with fried potato. |
| *Appearance* |  |
| Golden colour | The intensity of golden colour |
| Amount of dark edges | Amount of dark edges present, including burnt edges and skins. |
| Amount of Blemishes/black marks | Amount of blemishes/black marks present. |
| Greasy/oily | Visible greasiness or oiliness on the surface of the crisps. |
| Amount of Speckles | Amount of speckles of potato skin present over the whole sample. |
| *Texture* |  |
| Crispiness | The high-pitched sound produced when the product is bitten with the back teeth on the initial first bite. |
| Hardness | The force required to bite through the crisp. The harder the product the more force is required to bite through the sample. |
| Crunchiness | The amount of low-pitched noise and force with which the sample breaks or fractures during chewing with molars. |
| Greasiness | Greasy mouthfeel assessed during eating. |
| Thickness of cut | How thick the product feels whilst biting in the mouth from thin to thick. |
| Speed of breakdown | The amount of time for the food product to breakdown from the first chew to the swallow point from slow to fast. |
| Particles on fingers | The quantity of particles left on the fingers after touching the product. |
| Oiliness on fingers | The oiliness of the fingers after touching the product |
| *Flavour* |  |
| Initial Overall Flavour | The overall intensity of flavour (initial impact). |
| Overall Flavour during eating (DE) | The overall intensity of flavour assessed during eating |
| Saltiness (intial) | The intensity of the basic taste associated with Sodium Chloride upon initial impression. |
| Saltiness during eating (DE) | The intensity of the basic taste associated with Sodium Chloride overall during eating. |
| Sweetness | The intensity of the basic taste associated with sugar. |
| Oil | The intensity of the flavour associated with hot cooking oil. |
| Standard Potato | The intensity of flavour associated with a potato that has been fried in oil. |
| Reconstituted potato | The intensity of aftertaste associated with a potato that has been reconstituted, e.g. instant mashed potato or pringles. |
| Earthy | Intensity of flavour associated with the blemishes/skins which provides an earthy flavour note. |
| *Aftertaste* |  |
| Overall aftertaste intensity | The intensity of overall aftertaste. |
| Sweetness | The intensity of aftertaste associated with the basic taste sugar. |
| Saltiness | The intensity of aftertaste associated with the basic taste Sodium Chloride. |
| Oil | The intensity of aftertaste associated with cooked oil. |
| Standard potato | The intensity of aftertaste associated with a potato that has been fried in oil. |
| Reconstituted potato | The intensity of aftertaste associated with a potato that has been reconstituted, e.g. instant mashed potato or pringles. |
| Greasy mouthfeel | The oily film coating the mouth after swallowing. |

| Supplementary material 2a. Sensory descriptive analysis mean panel data (± standard deviation) and post-hoc test groupings for aroma and appearance attributes of 9 products. Different letters within a column show significant differences (p < 0.05). | | | | | | | | |
| --- | --- | --- | --- | --- | --- | --- | --- | --- |
|  | Aroma | | | Appearance | | | | |
| Product code^[[1]](#footnote-1)^ | Overall intensity | Oil | Fried potato | Golden colour | Amount of dark edges | Blemishes/black marks | Greasy/oily | Amount of speckles |
| P1 (STD) | 51.4 ± 10.5b | 38.1 ± 12.5b | 38.4 ± 12.0b | 38.4 ± 10.2bc | 13.6 ± 8.9de | 12.2 ± 7.9cd | 29.9 ± 13.1b | 10.9 ± 8.4c |
| P2 | 46.6 ± 10.1b | 38.4 ± 10.7b | 37.4 ± 8.6b | 37.4 ± 12.8c | 11.5 ± 8.9e | 14.8 ± 7.1bc | 38.2 ± 12.1b | 7.8 ± 9.6cd |
| P3 | 52.0 ± 15.6b | 39.7 ± 14.0b | 41.6 ± 12.7b | 41.6 ± 8.0bc | 22.7 ± 8.6c | 19.8 ± 8.8bc | 31.7 ± 11.5b | 28.4 ± 14.8b |
| High salt | 50.9 ± 12.2b | 38.4 ± 10.2b | 37.6 ± 10.8b | 37.6 ± 12.8bc | 20.4 ± 11.4cd | 17.2 ± 7.8bc | 32.7 ± 8.4b | 13.5 ± 10.4c |
| Medium salt | 52.0 ± 11.7b | 34.8 ± 11.3b | 37.3 ± 10.9b | 37.2 ± 8.9b | 31.1 ± 15.0b | 20.6 ± 13.0b | 31.9 ± 11.7b | 35.6 ± 21.1ab |
| Low salt | 51.1 ± 12.7b | 37.8 ± 10.4b | 37.4 ± 11.5b | 37.4 ± 10.1bc | 13.7 ± 9.0de | 17.5 ± 9.9bc | 35.2 ± 9.9b | 10.9 ± 10.1c |
| Hand-cooked | 75.3 ± 10.5a | 66.5 ± 18.3a | 44.7 ± 13.3a | 44.7 ± 8.3a | 65.8 ± 17.7a | 35.1 ± 15.9a | 57.4 ± 14.3a | 40.6 ± 16.2a |
| Crinkled | 53.8 ± 10.6b | 41.8 ± 12.4b | 40.9 ± 12.2b | 40.9 ± 13.7b | 22.1 ± 11.1c | 14.9 ± 9.4bc | 32.8 ± 14.0b | 16.2 ± 12.3c |
| Baked | 20.9 ± 9.2c | 11.2 ± 12.9c | 19.6 ± 13.0c | 19.6 ± 12.5d | 0.9 ± 2.2f | 0.2 ± 0.5d | 7.4 ± 7.2c | 1.0 ± 2.7d |

| Supplementary material 2b. Sensory descriptive analysis mean panel data (± standard deviation) and post-hoc test groupings for texture attributes for 9 products. Different letters within each column show significant differences (p < 0.05). | | | | | | | | |
| --- | --- | --- | --- | --- | --- | --- | --- | --- |
| Product code^[[2]](#footnote-2)^ | Crispiness | Hardness | Crunchiness | Greasiness | Thickness of cut | Speed of Breakdown | Particles on fingers | Oiliness on fingers |
| P1 (STD) | 62.9 ± 14.1abc | 36.8 ± 19.3e | 57.3 ± 13.9cd | 38.2 ± 14.5b | 33.3 ± 12.6d | 55.8 ± 15.9a | 40.0 ± 17.9a | 43.5 ± 19.4b |
| P2 | 62.6 ± 13.6abc | 37.3 ± 16.7e | 56.9 ± 13.7cd | 42.3 ± 16.7b | 33.2 ± 12.3d | 54.5 ± 15.3a | 37.6 ± 18.8a | 44.9 ± 16.7b |
| P3 | 64.9 ± 15.5abc | 47.5 ± 19.7cd | 60.9 ± 15.5cd | 41.0 ± 11.2b | 42.6 ± 12c | 50.6 ± 14.9a | 38.3 ± 18.3a | 45.2 ± 15b |
| High salt | 64.1 ± 10abc | 41.9 ± 20.7de | 62.9 ± 14.8c | 44.4 ± 15b | 35.9 ± 14.4cd | 53.7 ± 13.9a | 38.5 ± 17.9a | 43.9 ± 15.9b |
| Medium salt | 57.5 ± 14.2c | 40.6 ± 20.3de | 56.4 ± 16.6cd | 38.2 ± 14.4b | 39.4 ± 15.3cd | 55.0 ± 14.7a | 43.6 ± 19.5a | 45.6 ± 19.7b |
| Low salt | 58.6 ± 13.1bc | 34.9 ± 20.6e | 53.8 ± 16.1d | 38.2 ± 11.5b | 36.8 ± 13.6cd | 54.2 ± 15.1a | 38.0 ± 17.3a | 45.5 ± 18.7b |
| Hand-cooked | 67.2 ± 16.2ab | 78.1 ± 17.5a | 83.7 ± 7.2a | 61.9 ± 19.7a | 72.1 ± 15.1a | 30.2 ± 15.7b | 43.8 ± 16.1a | 57.8 ± 18.2a |
| Crinkled | 62.7 ± 11.4abc | 51.6 ± 15c | 64.8 ± 12.2cd | 40.9 ± 13.4b | 60.4 ± 16.7b | 48.2 ± 12a | 39.8 ± 21a | 45.5 ± 20b |
| Baked | 68.7 ± 10.2a | 60.7 ± 18.6b | 74.9 ± 12.4b | 7.1 ± 6.5c | 61.9 ± 14.9b | 27.8 ± 10.5b | 26.0 ± 17.6b | 12.8 ± 12.4c |

| Supplementary material 2c. Sensory descriptive analysis mean panel data (± standard deviation) and post-hoc test groupings for flavour attributes for 9 products. Different letters within each column show significant differences (P <0.05). | | | | | | | | | |
| --- | --- | --- | --- | --- | --- | --- | --- | --- | --- |
| Product code^[[3]](#footnote-3)^ | Initial Flavour Intensity | Saltiness flavour (Initial) | Overall flavour intensity (DE) | Saltiness flavour (DE) | Sweet flavour (DE) | Oil flavour (DE) | Potato flavour (DE) | Reconstituted potato flavour (DE) | Earthy flavour (DE) |
| P1 (STD) | 58.3 ± 12.3ab | 49 ± 11.8abc | 58.5 ± 12.7a | 50.6 ± 16.3ab | 7.4 ± 7.4b | 38 ± 14.9b | 46.5 ± 12.5a | 0 ± 0b | 0.5 ± 1b |
| P2 | 52.4 ± 13.9bc | 40.8 ± 13.4cd | 52.9 ± 13.8ab | 42.9 ± 14.1bc | 8.5 ± 7.6b | 39 ± 14.2b | 43.4 ± 13a | 0 ± 0b | 0.3 ± 0.7b |
| P3 | 57.4 ± 13.5ab | 46.1 ± 14.1abcd | 55.1 ± 13.7ab | 45.9 ± 17.9bc | 9.2 ± 9.5b | 38.4 ± 10.6b | 44.9 ± 12a | 0 ± 0b | 0.8 ± 2.1b |
| HIgh salt | 60.4 ± 9.4ab | 56.9 ± 10.1a | 60.6 ± 13.5a | 59.8 ± 14.5a | 6.1 ± 7.4b | 40.2 ± 13.3b | 45.7 ± 10.1a | 0 ± 0b | 0.8 ± 1.9b |
| Medium salt | 58.3 ± 11.1ab | 49.9 ± 16.5abc | 59.5 ± 10.9a | 53.1 ± 13.5ab | 7.9 ± 7.9b | 35.2 ± 12.3b | 45.1 ± 13.3a | 0 ± 0b | 1.2 ± 3.1b |
| Low salt | 51.7 ± 11.4bc | 35.2 ± 9.9de | 51.8 ± 13ab | 37.1 ± 15.7cd | 7.5 ± 7.5b | 35.2 ± 10.1b | 43.3 ± 12.8a | 0 ± 0b | 0.4 ± 1b |
| Hand-cooked | 61.6 ± 11.1a | 43.1 ± 7.4bcd | 61.3 ± 12.9a | 47.8 ± 10.7abc | 8.7 ± 11b | 58.8 ± 16.8a | 47.8 ± 12.2a | 0 ± 0b | 9.4 ± 11.2a |
| Crinkled | 57.9 ± 10ab | 52.3 ± 12.6ab | 61.1 ± 9.8a | 54.7 ± 11.3ab | 10.4 ± 8.8b | 35.8 ± 13.2b | 42.2 ± 11.4a | 0 ± 0b | 0.6 ± 1.7b |
| Baked | 44.2 ± 17.3c | 26.7 ± 11.5e | 47.5 ± 15.4b | 29.6 ± 16.4d | 49.8 ± 24.2a | 7 ± 5.2c | 0 ± 0b | 51.1 ± 27.5a | 0.1 ± 0.2b |

| Supplementary material 2d. Sensory descriptive analysis mean panel data (± standard deviation) and post-hoc test groupings for after taste attributes for 9 product. Different letters within each column show significant differences. | | | | | | | |
| --- | --- | --- | --- | --- | --- | --- | --- |
| Product code^[[4]](#footnote-4)^ | Overall Aftertaste Intensity | Sweet aftertaste | Saltiness aftertaste | Oil aftertaste | Potato aftertaste | Reconstituted Potato after taste | Greasy mouthfeel |
| P1 (STD) | 48.1 ± 16.9ab | 5 ± 5.9b | 33 ± 18.6ab | 32.9 ± 18.1b | 35.5 ± 13.2ab | 0 ± 0b | 35.7 ± 19.7b |
| P2 | 43.6 ± 16b | 4.7 ± 5.6b | 28.6 ± 17.1abc | 35.4 ± 18.5b | 32.5 ± 16.1b | 0 ± 0b | 37.7 ± 20.6b |
| P3 | 47.2 ± 12.5ab | 5.7 ± 7.2b | 28.7 ± 13.9abc | 32.9 ± 12.2b | 32.6 ± 12.9b | 0 ± 0b | 36.7 ± 14.5b |
| High salt | 47.8 ± 14ab | 4.1 ± 6.5b | 39.3 ± 16.8a | 34.5 ± 16.3b | 34.9 ± 12.9ab | 0 ± 0b | 37.6 ± 18.6b |
| Medium salt | 49.5 ± 14.9ab | 5.2 ± 6.9b | 35.6 ± 17.9ab | 33.4 ± 14.1b | 35.8 ± 12.7ab | 0 ± 0b | 37.4 ± 14b |
| Low salt | 43.9 ± 16.6b | 4.8 ± 6.2b | 24.5 ± 17.1bc | 33.2 ± 15.4b | 33.3 ± 13.7b | 0 ± 0b | 33.3 ± 18.7b |
| Hand-cooked | 54.5 ± 15.9a | 7.4 ± 9.4b | 32.7 ± 15.7ab | 47.9 ± 17.7a | 40.2 ± 13.6a | 0 ± 0b | 51.2 ± 19.5a |
| Crinkled | 47.4 ± 14.7ab | 6.9 ± 8.2b | 35.1 ± 16.1ab | 32.1 ± 10.6b | 35.5 ± 12.5ab | 0 ± 0b | 37.2 ± 16b |
| Baked | 42.9 ± 15.2b | 38.9 ± 15.1a | 20.3 ± 15.4c | 4.1 ± 3.9c | 0 ± 0c | 40.1 ± 30.3a | 4.5 ± 6.8c |

Supplementary material 3. Dendrogram of agglomerative hierarchical clustering of consumers (n=93).


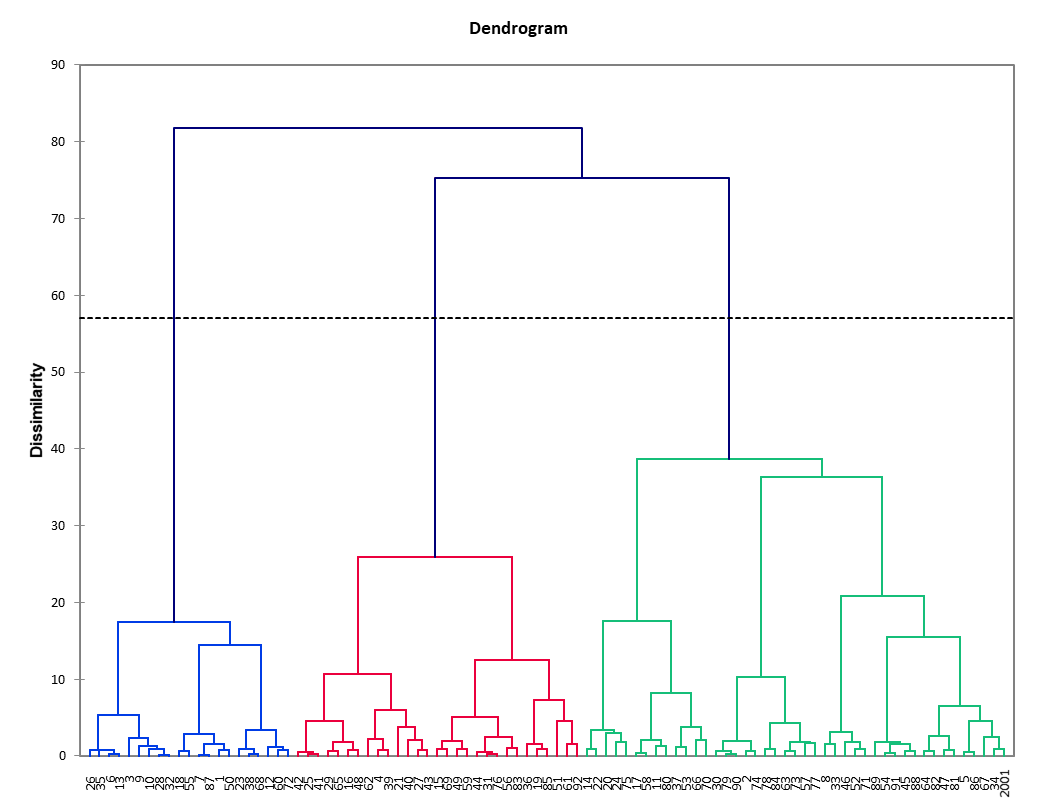


Supplementary material 4. Just-about-right (JAR) responses^[[5]](#footnote-5)^ for each product^[[6]](#footnote-6)^ for each cluster group shown as a percentage.


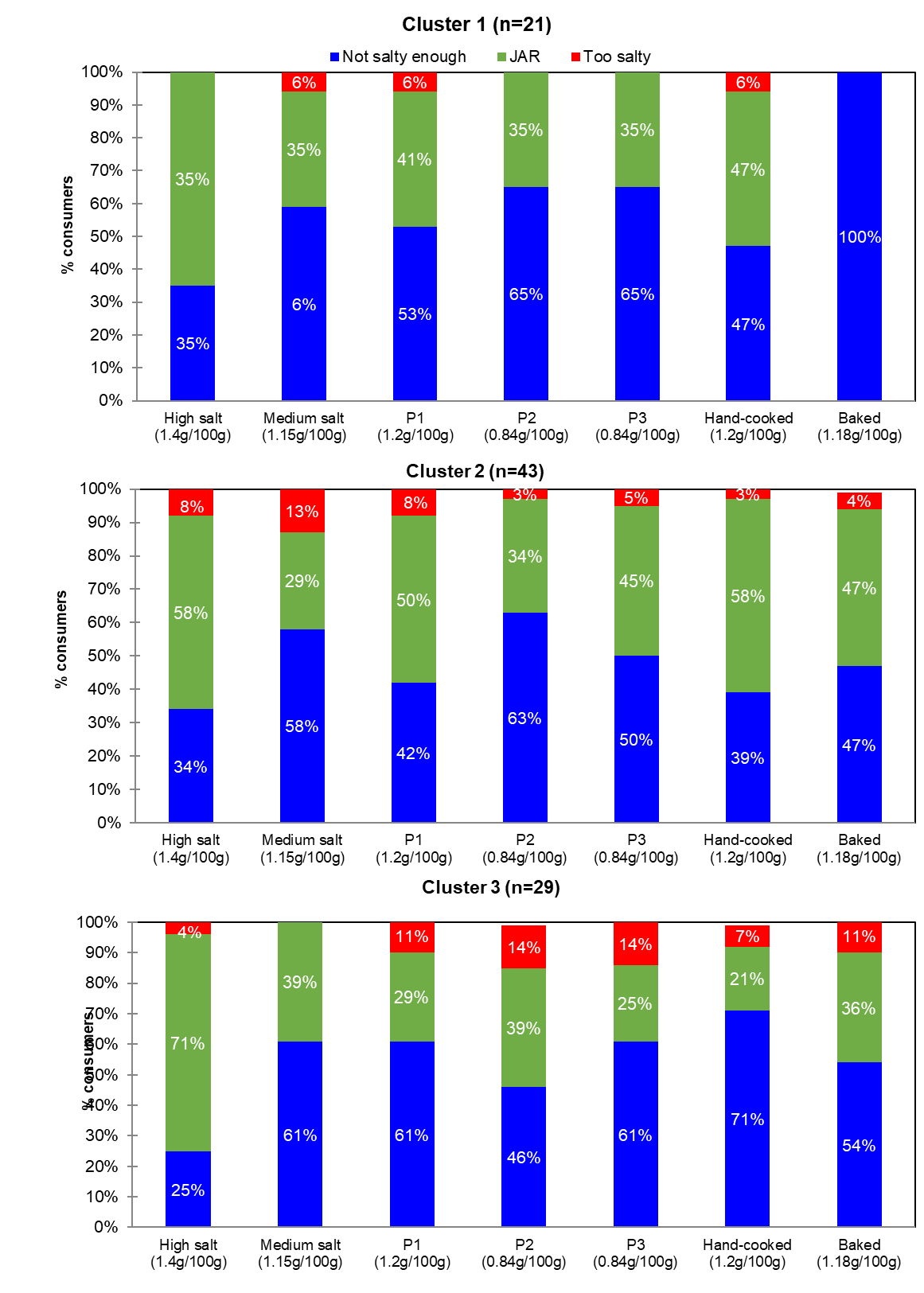


| Supplementary material 5. Demographic, behavioural characteristics and potato crisp consumption habits for each mouth behaviour group | | | | |
| --- | --- | --- | --- | --- |
|  | Frequency response (%) | | | |
|  | Chewer  (n=20) | Cruncher  (n=33) | Smoosher  (n=9) | Sucker  (n=21) |
| **Gender** |  |  |  |  |
| Female | 45% | 52% | 56% | 52% |
| Male | 55% | 48% | 44% | 48% |
| **Age** |  |  |  |  |
| 18-24 | 20% | 21% | 0% | **5%** |
| 25-34 | 10% | 9% | 11% | 19% |
| 35-44 | 25% | 24% | 11% | **5%** |
| 45-54 | 25% | 24% | 44% | 29% |
| 55-64 | 20% | 21% | 33% | 43% |
| **Consumption habits (any flavour)** |  |  |  |  |
| Less than once a week | 5% | 12% | 0% | 10% |
| Once a week | 10% | 0% | 22% | 5% |
| 2-4 times a week | 50% | 58% | 56% | 71% |
| Once a day or more | 35% | 30% | 22% | 14% |
| **Consumption habits (ready salted)** |  |  |  |  |
| Less than once a week | 5% | 6% | 0% | 5% |
| Once a week | 25% | 24% | 44% | 38% |
| 2-4 times a week | 55% | 42% | 33% | 43% |
| Once a day or more | 5% | 3% | 11% | 5% |
| **Normal commercial crisp consumption** |  |  |  |  |
| Walkers ready salted | 95% | 76% | 89% | 76% |
| Walkers Hint of Salt | 10% | 18% | 22% | 10% |
| Supermarket own brand ready salted crisps | 45% | 36% | 67% | 48% |
| Walkers oven baked sea salt | 40% | 52% | 33% | 43% |
| Kettle original sea salt | 45% | 45% | 44% | 43% |
| Pringles original salted | 65% | 73% | 56% | 86% |
| Smiths original crinkle cut salted | 5% | 9% | 11% | 19% |
| Hula Hoops original salted | 65% | 64% | 56% | 71% |
| Chipstix ready salted | 15% | 3% | 11% | 14% |
| McCoys Ridge Cut salted | 55% | 52% | 56% | 48% |
| Originally Smiths Walkers salt and shake | 20% | 18% | 22% | 14% |
| Tyrells Lightly salted | 20% | 30% | 22% | 29% |
| Walkers crinkles simply salted | 30% | 30% | 22% | 33% |
| Popchips sea salted | 20% | 3% | 0% | 5% |
| Pom Bear Original | 30% | 30% | 11% | 29% |
| Walkers French fries ready salted | 55% | 36% | 33% | 43% |
| Hula hoops puft salted | 20% | 30% | 22% | 14% |

Supplementary material 6. Mean liking scores of each product^[[7]](#footnote-7)^ per mouth behaviour group for each liking attribute. Error bars represent standard error. Different letters indicate a significant difference between product (p < 0.05).


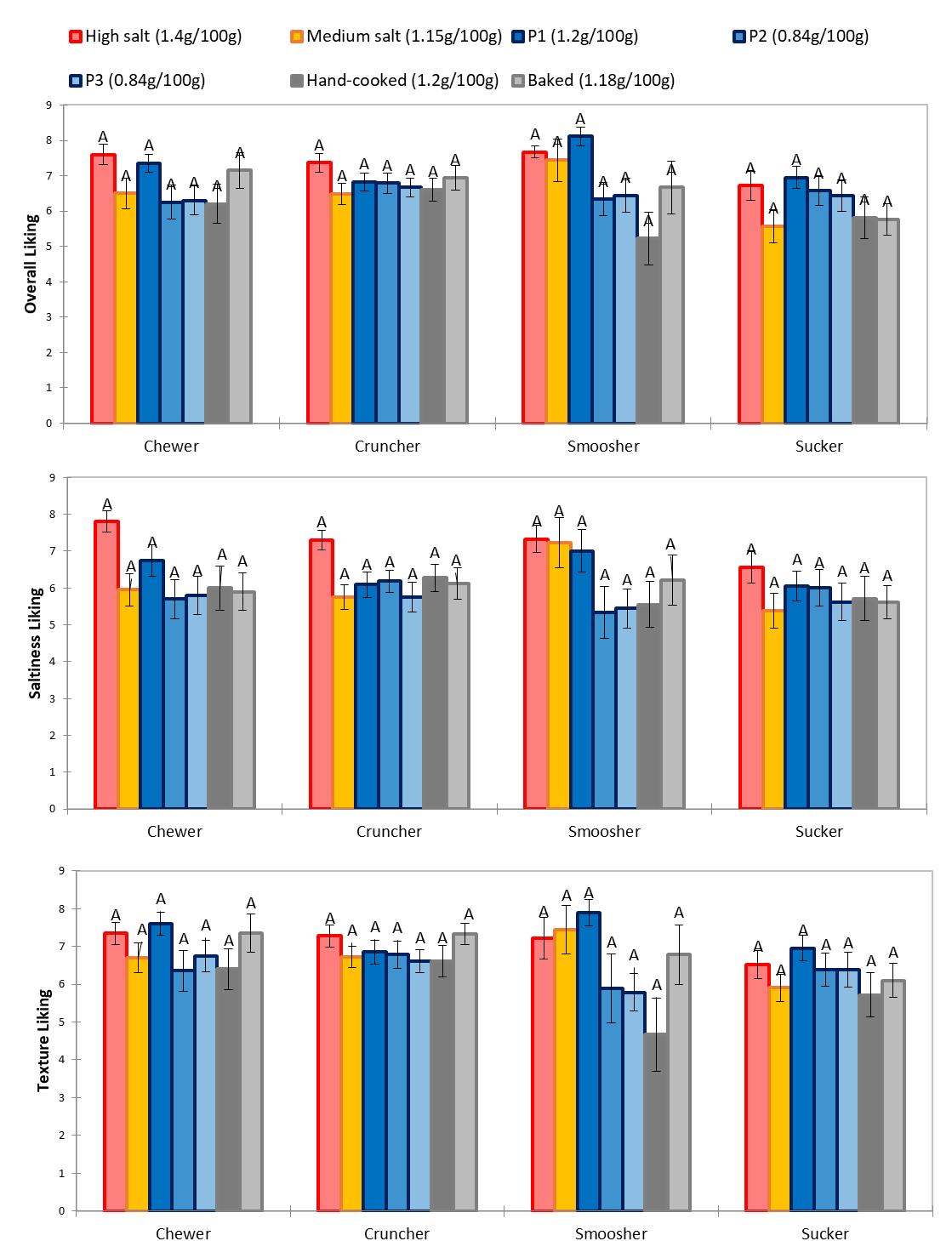


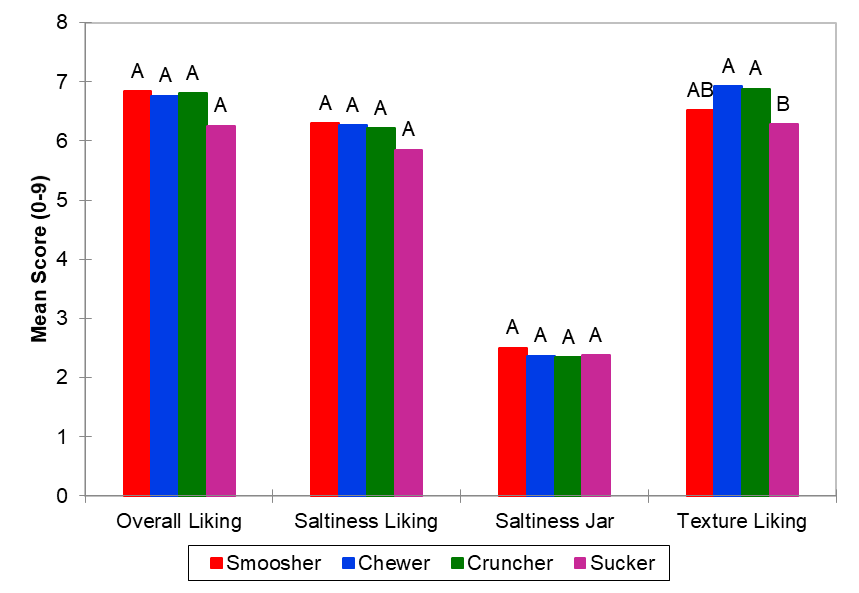
Supplementary material 7. Mean liking scores for each liking attribute for each mouth behaviour phenotype group. Different letters within each category determine a significant difference (p < 0.05)

1. Product details are outlined in Table 1. [↑](#footnote-ref-1)
2. Product details are outlined in Table 1. [↑](#footnote-ref-2)
3. Product details are outlined in Table 1. [↑](#footnote-ref-3)
4. Products are detailed in Table 1. [↑](#footnote-ref-4)
5. Responces “not quite salty enough” and “not at all salty enough” were grouped into the “not salty enough” category and responces “too salty” and “far too salty” were grouped into the “too salty” category [↑](#footnote-ref-5)
6. Potato crisp products include prototypes (P1, P2, P3) with the rest being commercial products detailed in the text. P1 was made using 1.2g of 106-425 µm NaCl salt per 100g of unsalted potato crisps. P2 and P3 are 30% reduced NaCl salt products compared to P1 made using <106 µm NaCl salt or SODA-LO® salt microspheres respectively. [↑](#footnote-ref-6)
7. Potato crisp products include prototypes (P1, P2, P3) with the rest being commercial products detailed in the text. P1 was made using 1.2g of 106-425 µm NaCl salt per 100g of unsalted potato crisps. P2 and P3 are 30% reduced NaCl salt products compared to P1 made using <106 µm NaCl salt or SODA-LO® salt microspheres respectively. [↑](#footnote-ref-7)
